# Neurodevelopmental Trajectories in Preterm Neonates: Integrating Neuroimaging Modalities with Clinical Neurological Outcomes

**DOI:** 10.3390/diagnostics16091356

**Published:** 2026-04-30

**Authors:** Andreea Ioana Necula, Roxana Pavalache-Stoiciu, Larisa Nicoleta Andrasoaie, Al Jashi Isam

**Affiliations:** 1 Doctoral School, “Carol Davila” University of Medicine and Pharmacy, 020956 Bucharest, Romania; andreea-ioana.necula@drd.umfcd.ro; 2 Department of Neonatology, MedLife Medical Park, 013695 Bucharest, Romania; 3 Faculty of Medicine, University Titu Maiorescu, 040441 Bucharest, Romania; aljashiisam@yahoo.com

**Keywords:** preterm, neurodevelopment, head ultrasound, MRI, General Movements

## Abstract

This narrative review aims to demonstrate how integrating neuroimaging with functional assessments and standardized protocols enhances the identification of long-term motor and psychiatric risks. This review synthesized 12 studies from the last 5 years. The analysis focused on preterm infants (<37 weeks’ gestational age) and evaluated the correlation between neuroimaging (head ultrasound (HUS) and Magnetic Resonance Imaging (MRI)), head circumference (HC), and functional assessments like Prechtl General Movements (GMs). While HUS remains the primary bedside tool, its sensitivity for subtle, non-cystic white matter injury is limited compared to MRI. Both modalities demonstrate high negative predictive values at term-equivalent age (TEA) for excluding severe motor deficits. Structural markers, including increased ventricular midbody size, immature gyration, and bilateral lesion laterality, were strongly associated with Cerebral Palsy (CP) and gross motor delays. Furthermore, TEA assessments provided superior prognostic accuracy compared to early neonatal scans. Optimal outcomes were linked to the integration of neuroimaging with functional assessments (GMs) and reliable parental support to ensure follow-up compliance. A tiered HUS/MRI protocol combined with routine GMs assessment enables precise prognostic counseling. Correlating TEA imaging with long-term findings necessitates follow-up beyond 24 months.

## 1. Introduction

The remarkable increase in survival rates for infants with very low birth weight (VLBW) has presented a significant clinical challenge: the high prevalence of long-term neurodevelopmental morbidities. Survivors remain at significant risk for a range of disabilities, including Cerebral Palsy (CP), behavioral disorders, and impaired academic achievement. To improve neonatal intensive care and evaluate new therapies, it is essential to clearly define the structural and functional brain abnormalities that predispose these infants to such difficulties.

Longitudinal data indicate a clear severity gradient. Infants with high-grade intraventricular hemorrhage (IVH) and associated hydrocephalus, particularly those requiring surgical shunting, demonstrate the highest risk for significant morbidity across neuropsychological function and academic skills [1].

Serial monitoring of the intracranial ventricles is a clinical priority. Standardized reference ranges for the anterior horn width and thalamo-occipital distance have validated head ultrasound (HUS) as a reliable tool for tracking enlargement, regardless of the infant’s sex or head position [2]. While HUS is the standard bedside method for diagnosing major hemorrhages and ventriculomegaly, its sensitivity for detecting subtle injuries remains a subject of ongoing studies.

Early research highlighted that while standard HUS measures are reliable for tracking the growth of the corpus callosum and basal ganglia, they may lack the precision needed to quantify subtle cortical depth or extracerebral space changes [3]. Qualitative studies using term-equivalent Magnetic Resonance Imaging (MRI) have demonstrated superior sensitivity, revealing a high prevalence of moderate to severe non-cystic white matter (WM) abnormalities that were frequently missed by traditional ultrasound. These diffuse injuries are often linked to perinatal risk factors such as infection or hypotension requiring inotropic support, underscoring the need for advanced imaging to define the full nature of cerebral abnormalities [4].

The predictive power of neuroimaging is significantly improved when integrated with functional assessments. The evaluation of General Movements (GMs) through Prechtl analysis has developed as a highly sensitive tool for the early detection of neurological risk. GMs show strong agreement with both ultrasound findings and clinical exams, but more importantly, they can identify potential dysfunction in high-risk infants whose early HUS appears normal [5].

Recent efforts, such as the standardization of neonatal assessment in Romania, have implemented comprehensive examination protocols like the Amiel-Tison method. This progress has shifted research priorities toward identifying long-term psychiatric risks, including autism spectrum disorder, ensuring that the follow-up of former preterm infants extends beyond motor deficits to include psychological and behavioral outcomes [6].

While neuroimaging technologies have progressed, standardized protocols and validated prognostic tools for preterm infants are not yet fully established. This narrative review integrates recent findings to propose a structured, evidence-based stratification strategy.

The present review has a dual objective. First, it aims to evaluate the performance of HUS and MRI in the early detection and classification of brain injury in preterm infants, including WM injury, hemorrhage, and structural abnormalities. Second, it examines the prognostic value of these imaging modalities in predicting long-term neurodevelopmental outcomes (CP, motor and cognitive impairments).

## 2. Materials and Methods

A literature review was conducted to synthesize current evidence regarding the correlations between neonatal neuroimaging, anthropometric clinical markers, and long-term neurobehavioral development in preterm infants. The search was performed across primary biomedical databases, including PubMed, Scopus, and Web of Science. The literature was identified using a targeted keyword-based search strategy, incorporating terms such as “preterm neonate”, “VLBW”, “HUS”, “MRI”, “neurodevelopmental outcome”, and “GMs”.

A total of 12 articles, comprising prospective and retrospective cohort studies, reviews, and clinical commentaries, were selected for analysis.

The inclusion criteria included:

- Population: preterm infants, ranging from extremely preterm (<28 weeks gestational age) to late preterm (<34–37 weeks gestational age).
- Diagnostic tools: HUS, MRI, or head circumference (HC) measurements.
- Assessments: correlate imaging findings with standardized functional tools, such as GM Prechtl analysis.
- Timeframe: studies from the last 5 years.
- Studies were excluded if they met any of the following:
- Technical standards: use of outdated imaging technology or non-standard protocols.
- Publication type: case reports, conference abstracts, or non-English language papers.

We focused on a limited number of articles because they provided the most reliable and up-to-date data. By keeping the selection small, we ensured that all the studies used similar imaging methods and the same types of physical exams, making the results easier to compare.

**Figure 1:**
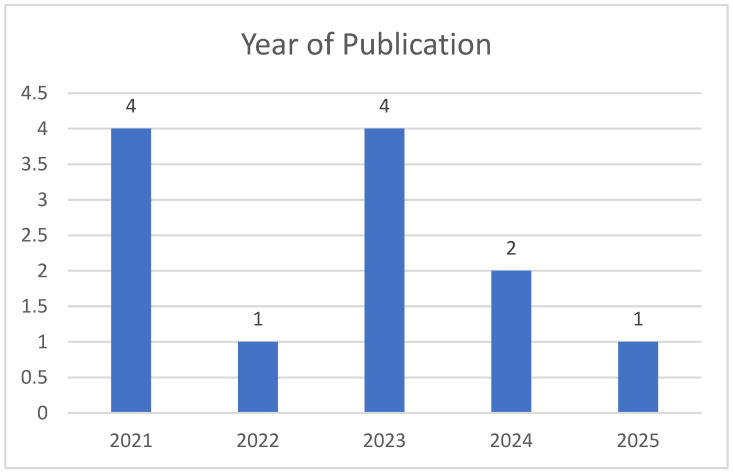
Distribution of studies by year of publication.

The chronological distribution of the 12 included studies reflects a strong emphasis on contemporary research, with the majority published within the last 3 years (Figure 1). Specifically, the literature synthesis comprises 4 studies from 2021 (Kwong, A.K. et al. [7], Guillot, M. et al. [8], Inder, T.E. et al. [9], Zhang, X.H. et al. [10]), 1 from 2022 (Helderman, J. et al. [11]), 4 from 2023 (Kumar, N. et al. [12], McLean, G. et al. [13], Toma, A.I. et al. [14], Chevallier, M. et al. [15]), 2 from 2024 (Mayrink, M.L.D.S. et al. [16], Toma, A.I. et al. [17]), and 1 recent publication from 2025 (Necula, A.I. et al. [18]), ensuring the integration of the most current clinical insights and neuroimaging advancements.

**Figure 2:**
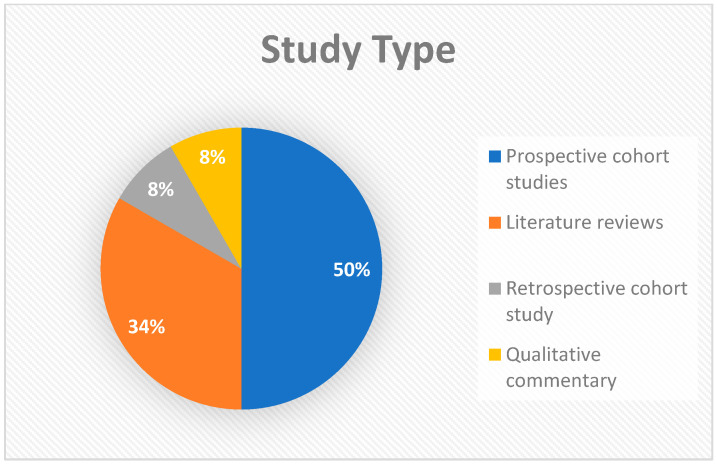
Proportion of study types within the review.

The 12 included studies comprised a diverse methodological mix (Figure 2): 6 prospective cohort studies (Zhang, X.H. et al. [10], Helderman, J. et al. [11], Kumar, N. et al. [12], Toma, A.I. et al. [14], Mayrink, M.L.D.S. et al. [16], Toma, A.I. et al. [17]), 4 literature reviews (Guillot, M. et al. [8], Inder, T.E. et al. [9], Chevallier, M. et al. [15], Necula, A.I. et al. [18]), 1 retrospective cohort study (McLean, G. et al. [13]), and 1 qualitative commentary (Kwong, A.K. et al. [7]). This combination ensures a comprehensive synthesis of high-level primary evidence and expert clinical consensus.

## 3. Results

The review of the 12 included studies (summarized in Table 1), ranging from prospective cohorts in Brazil and Romania to comprehensive reviews from Canada and the USA, reveals an integrated approach to predict neurodevelopment in preterm infants. These studies evaluate clinical markers, such as HC, alongside advanced neuroimaging and functional neurological assessments to establish early prognostic indicators. The evidence consistently highlights that while survival rates for very preterm infants have improved, the identification of subtle WM injuries and the timing of assessments remain critical challenges for clinical practice.

### 3.1. Imaging Modalities and Diagnostic Sensitivity (Table 2)

The current literature reflects a developing trend in the comparative utility of neonatal neuroimaging. While HUS remains the primary bedside tool due to its safety and cost-effectiveness [11,15], its sensitivity is increasingly studied. A literature review observed a shift from cystic WM injury toward a more discrete, non-cystic pattern that HUS often fails to detect, whereas MRI provides superior visualization of these diffuse anomalies [8]. This is further supported by a review, which recommended MRI for its high sensitivity in identifying cerebellar hemorrhages and WM maturation [9]. Despite these technical advantages, the predictive value for long-term functional outcomes remains complex. Both HUS and MRI demonstrate high negative predictive values at term-equivalent age (TEA), meaning a normal scan is highly reliable for predicting the absence of severe motor impairment [8,13].

**Table 2:** Comparative analysis of HUS and MRI in predicting neurodevelopmental outcomes in preterm infants (CP, Cerebral Palsy; HUS, head ultrasound; MRI, Magnetic Resonance Imaging; PVL, periventricular leukomalacia; and WM, white matter).

Metric	HUS	MRI	Source
CP	High specificity (96–98%), but low sensitivity (12–27%)	PVL: 60–67%. Superior for detecting subtle WM injury	Inder, T.E. et al. [9], McLean, G. et al. [13]
Cognitive Outcomes	Low correlation; poor at identifying diffuse injury	High negative predictive value. Low positive predictive value	Guillot, M. et al. [8], Inder, T.E. et al. [9]
Severe Lesions	Optimal for Grade III/IV IVH and cystic PVL	Equivalent to HUS but offers more anatomical detail	Zhang, X.H. et al. [10], Chevallier, M. et al. [15]
Subtle Injuries	Often missed unless a specific scoring system is used	Superior sensitivity for non-cystic WM injury and cerebellar bleeds	Guillot, M. et al. [8], Inder, T.E. et al. [9]
Early Predictors	Early scans (first 2 weeks) have lower predictive power	Not typically used in the acute phase for routine prognosis	Zhang, X.H. et al. [10], Helderman, J. et al. [11]

### 3.2. Anthropometric and Structural Predictors of Neurodevelopment

Beyond advanced imaging, physical markers such as HC provide significant prognostic value. A prospective cohort study established that HC growth, particularly during the fast growth period between discharge and 1 month corrected age, serves as a valuable clinical marker that correlates positively with cognitive, motor, and language at 18 months [16]. When examining internal structures, birth weight acts as a primary protective factor, while high-grade hemorrhages on initial scans were the strongest predictors of impaired development and mortality [10]. Furthermore, structural abnormalities in the posterior region of the corpus callosum have been associated with disrupted sensory–motor integration and cognitive deficits in school-aged children, as demonstrated by Lubián-Gutiérrez et al. (2024) [19] and supported by additional studies [18]. While a prospective cohort study mentioned birth weight as a primary protective factor [10], another prospective study reported a higher incidence of abnormal neuroimaging in babies with birth weights > 2 kg (44.44%) [12].

### 3.3. Lesion Characteristics and Motor Outcomes

The relationship between specific brain lesions and motor dysfunction, such as CP, is well-documented but complex. A prospective cohort study found that abnormal gross and fine motor acquisitions at 24 months are significantly linked to increased ventricular midbody size and immature gyration folding at TEA [17]. Similarly, a review reported that 83% to 91% of infants with cystic periventricular leukomalacia (PVL) eventually develop CP [9]. Regarding intraparenchymal hemorrhages, laterality has been reported as a more consistent predictor of disability than specific anatomical location, with bilateral lesions significantly associated with a higher risk of severe CP compared to unilateral ones, as demonstrated by Maitre et al. (2009) [20] and further supported by the subsequent literature [15].

### 3.4. Functional Assessments and the Role of Timing

The timing of assessment is critical for diagnostic accuracy. A prospective cohort study [10] and a retrospective study [13] concluded that early scans often lack correlation with long-term outcomes, whereas scans performed at TEA provide a more accurate prognostic timeline. To improve sensitivity, formalized scoring systems that include subtle markers should be implemented. Imaging findings are frequently triangulated with functional exams. Atypical GMs correlate with HUS markers of WM volume loss [14].

Finally, the clinical translation of these findings depends on parental engagement. Without adequate emotional and medical support, parents may struggle to comply with follow-up schedules necessary for early neurological diagnosis and intervention [7].

## 4. Discussion

The relationship between neonatal imaging and neurodevelopmental outcomes can be visualized as a multi-step process. First, optimization of imaging modality and timing is essential to accurately detect brain injury. Second, these imaging findings must be converted into reliable and quantifiable markers of neurological impairment. Third, these markers serve as the basis for predicting long-term neurodevelopmental outcomes (motor, cognitive, and behavioral). These interconnected steps highlight the importance of both diagnostic accuracy and prognostic relevance when evaluating imaging techniques in preterm infants.

The correlation between neonatal neuroimaging and long-term neurologic outcomes in preterm infants has evolved from a focus on major hemorrhages to the detection of subtle WM and cerebellar injuries. While early HUS detects major hemorrhages, near-term MRI is superior at identifying cerebellar lesions and WM abnormalities. These findings were independently associated with neurodevelopmental impairment and gross motor delay at 18–22 months [21].

Modern neuroimaging shows that major cystic lesions have decreased in incidence, but diffuse WM injury, which often remains undetected by early HUS, is highly prevalent in extremely preterm infants and correlates with long-term cognitive deficits [22]. In VLBW infants, HUS’s high reliability for cystic injury detection is neutralized by its inability to identify non-cystic WM injury, the more prevalent and common form of WM pathology in modern neonatology [23].

Recent investigations into subcortical development have utilized advanced shape and structural correlation of MRI analyses to map the effects of preterm birth on the thalamus. Moving beyond global volumetric assessments, these studies identified a heterogeneous vulnerability within the thalamic subregions. Furthermore, analyses reveal an abnormal increase in structural association between the thalamus and the insula in preterm neonates [24]. These findings suggest that prematurity drives a complex reorganization of thalamic microstructure and connectivity.

Since both HUS and MRI serve as pillars of neonatal neurology, their roles are increasingly defined by a stepped, stratified approach to risk assessment. Due to the high prevalence of white and gray matter deficits in very preterm neonates, a more comprehensive assessment for neuroimaging is required. A specific MRI scoring tool would provide a more objective and systematic classification of brain injury and maturation compared to previous diagnostic standards [25].

While a normal HUS is highly reassuring because of a positive predictive value of 99% for normal neuromotor development, a “normal” scan does not guarantee a normal outcome [26,27]. A sinocortical width > 3.5 mm at TEA was shown to be an independent risk factor for developmental delay, particularly in the gross motor domain [28]. The site of injury is a critical determinant. Recent findings address the diagnostic gap in preterm infants who lack severe structural injury yet still develop neurodevelopmental deficits. Their findings suggest that a structurally normal MRI does not guarantee functional integrity. Favorable outcomes appear to rely on a specific compensatory hyper-connectivity within thalamo-limbic and thalamo-basal ganglia circuits. The failure to generate this compensatory response serves as a silent marker of pathway dysmaturation, indicating that functional connectivity analysis is essential to detect latent risks in infants [29].

In very preterm infants, the extent of periventricular hemorrhagic infarction and its associated severity score served as reliable predictors of adverse outcomes, specifically regarding gross motor impairment and neonatal mortality [30]. Periventricular hemorrhagic infarction involving the trigone or parieto-occipital region carries the highest risk for motor deficits [31]. However, laterality also plays a key role. Bilateral lesions significantly increase the risk of severe CP compared to unilateral ones [15].

Historically, HUS was criticized for its inability to detect non-cystic white and gray matter injuries that may cause cognitive deficits [32]. However, recent data suggests that the limitation may lie in the lack of standardized scoring. When a rigorous, quantitative scoring system is applied at TEA, HUS’s predictive power rivals that of MRI [33]. A study further emphasized that formalizing these scoring systems to include subtle markers like corpus callosum thinning is essential for prognostic accuracy [13]. Recent cohort studies have specifically linked abnormal ventricular midbody size and immature gyration folding at TEA to impaired gross and fine motor acquisitions at 24 months [17]. Serial scanning is essential to capture cystic lesions before they collapse, and follow-up must extend to school age to accurately track the preterm infant’s true neurodevelopmental trajectory [34].

To balance diagnostic precision with healthcare costs, a screening protocol is recommended. A suggestion would be prioritizing TEA MRI for high-risk infants, while serial HUS is considered sufficient for lower-risk infants [35]. The timing of the scans is crucial, serial scanning being essential to prevent false negative cystic lesions that collapse over time [36], while early scans often lack correlation with long-term outcomes compared to scans performed at TEA [8,10]. Many cognitive and academic impairments do not manifest until school age. Furthermore, research must extend beyond the standard 18 to 24 months follow-up in order to capture the true neurodevelopmental trajectory of the preterm infant [36,37].

It must be taken into consideration that MRI is more sensitive than HUS for detecting diffuse WM gliosis and cerebellar injury [38]. While traditional neuroimaging in preterm neonates has heavily focused on supratentorial structures, a review highlights that the severity of prematurity disrupts cerebellar development via maturation failure. Crucially, these cerebellar disruptions are linked to a spectrum of long-term deficits, including cognitive, language, and behavioral impairments. This evidence suggests that excluding the cerebellum from neurodevelopmental models may underestimate a key contributor to the complex neurocognitive patterns observed in preterm survivors [39].

Beyond structural damage, recent functional MRI studies have shown that preterm infants display altered temporal signal complexity at TEA, particularly in motor and visual networks, which serves as a biomarker for developmental delay even when structural scans appear normal [40]. Complementing these findings, longitudinal research provides compelling evidence for the use of serial functional MRI, demonstrating that the rate of increase in hippocampal activity during early infancy is a robust predictor of 18-month cognitive and motor outcomes [41].

While over 90% of infants from one study showed normal cognitive function at 1 year, a significant portion (roughly 1/3) who were classified as “not delayed” at age 1 were later found to have delays at age 2, likely due to the increasing cognitive demands of testing. They found that assessments at 2 years corrected age are far more rigorous predictors of preschool cognitive development (age 5) than those at 1 year, although a combination of both assessments provides the highest predictive value. These findings highlight the complexity of developmental trajectories and support the necessity of comprehensive, long-term follow-up programs that extend to preschool age to capture evolving patterns of impairment [42].

The highest prognostic precision was shown to be achieved when quantitative structural markers, such as interhemispheric distance and deep gray matter integrity, are interpreted alongside functional milestones assessed during the 3-to-5-month post-term window. This targeted approach allows the identification of infants on abnormal neurodevelopmental trajectories who require immediate therapeutic support [43].

By school age (around 8 years), extremely preterm children exhibit significantly higher rates of intellectual impairment, as well as poorer executive function, academic achievement, and motor coordination compared to term-born controls. Despite medical advances, approximately 20–40% of extremely preterm survivors face major neurodevelopmental disabilities, underscoring the critical need for long-term monitoring and new intervention strategies that extend into school years [44].

Cognitive, behavioral, and academic impairments, such as Attention Deficit–Hyperactivity Disorder (ADHD), executive dysfunction, and social struggles, often remain latent until the child faces a complex environment of school [45].

Clinical context remains a top priority. Factors such as prolonged mechanical ventilation can lead to hypocapnia and subsequent WM injury, necessitating that neuroimaging be interpreted alongside clinical history [46]. While birth weight is generally a primary protective factor, some data show a surprisingly high incidence of abnormal neuroimaging in neonates with birth weights > 2 kg (44.44%), suggesting that large preterm infants still require vigilant screening [10]. Moreover, in term neonates, evidence suggests that cerebral size is similar across different ethnic groups, supporting universal reference ranges [47].

Recent systematic reviews indicate that integrating multimodal data, combining MRI with clinical history via machine learning algorithms, significantly outperforms unimodal models in predicting neurodevelopmental impairment [48].

Emerging evidence suggests that the neurodevelopmental trajectory of the preterm brain is not solely determined by biological injury but is also shaped by extaruterine environment. A recent study demonstrated that the duration of skin-to-skin holding in the neonatal intensive care unit (NICU) was significantly associated with the microstructural organization of fronto-limbic white matter tracts, specifically the cingulum and anterior thalamic radiations, in infants born under 32 weeks of gestation. Future prognostic models must take into consideration environmental exposure (intervention frequency and duration) to accurately map the development curve [49].

A broader review cautions that while Artificial Intelligence (AI) offers transformative potential for integrating complex NICU data, including imaging and clinical metrics to predict outcomes, the current evidence base is inconsistent because of a lack of external validation and integrated implementation strategies, limiting direct bedside application [50].

Neonatal brain development is shaped by the balance of injury, quality of clinical care, and early detection. To eliminate diagnostic gaps, it is essential to combine the routine utility of HUS with the precision of MRI to fully capture deep gray matter and white matter maturation. Integrating both imaging modalities with standardized neurodevelopmental assessments provides a more reliable pathway to connect neonatal findings with school-age outcomes.

## 5. Conclusions

In conclusion, neonatal neuroimaging has transitioned from identifying gross hemorrhages to detecting subtle WM and cerebellar injuries. While MRI remains the gold standard, a stratified approach for HUS is increasingly viable. By implementing standardized, quantitative scoring at TEA, the prognostic accuracy of HUS can rival that of MRI. Predictive precision depends heavily on the site and laterality of injury, as well as the consideration of clinical variables such as birth weight and HC.

Future protocols prioritize TEA-specific imaging and extended follow-up beyond 24 months to accurately capture the long-term neurodevelopmental evolution of the preterm infant.

We recommend a tiered imaging protocol: routine HUS for all preterm neonates, with MRI at TEA reserved for high-risk infants. Standardized functional tests, such as GMs, might be used following further research. Additionally, follow-up must extend beyond 24 months to identify long-term neurodevelopmental outcomes.

## Figures and Tables

**Table 1:** Key findings from included studies.

Reference	Title	Year	Study Type	Country	Population	Key Findings
Kwong, A.K. et al. [7]	“Early neurodevelopmental screening: Parent perspectives from the neonatal intensive care unit”	2021	qualitative commentary	USA	In total, 19 parents in Level III and Level IV NICU	To ensure follow-up compliance, parents require structured emotional and professional support; a lack of information prevents parents from understanding the necessity of long-term neurological re-examinations
Guillot, M. et al. [8]	“Comparative performance of head ultrasound and MRI in detecting preterm brain injury and predicting outcomes: a systematic review	2021	literature review	Canada	preterm neonates	A shift from overt cystic WM injury to diffuse, non-cystic patterns has been captured by MRI; while HUS and MRI reliably predict motor deficits (CP), predicting cognitive outcomes remains a significant challenge; TEA imaging yields high negative predictive value, making it highly effective at ruling out severe impairment
Inder, T.E. et al. [9]	Neuroimaging of the Preterm Brain: Review and Recommendations”	2021	literature review	USA	preterm neonates	Accurate prognosis depends on matching the neuropathology to the modality: HUS is effective for apparent hemorrhagic lesions, but MRI is superior for detecting cerebellar hemorrhages and diffuse WM injury; MRI growth metrics provide objective measures of brain development that help characterize the impact of preterm birth on future cognitive and behavioral outcomes
Zhang, X.H. et al. [10]	“Predicting the developmental outcomes of very premature infants via ultrasound classification A CONSORT- clinical study”	2021	prospective cohort study	China	In total, 129 very preterm infants (<28 weeks GA)	Birth weight is the most significant protective factor against poor outcomes; while early scans do not correlate with long-term development, serial HUS classification ending at TEA demonstrates strong associations with mental and psychomotor development indices
Helderman, J. et al. [11]	“Association of abnormal findings on neonatal cranial ultrasound with neurobehavior at neonatal intensive care unit discharge in infants born before 30 weeks’ gestation”	2022	prospective cohort study	USA	In total, 704 infants (<30 weeks GA)	WM injury detected within the first 2 weeks of life is significantly associated with poor attention and movement quality at discharge; early HUS serves as a safe, cost-effective bedside triage tool to identify infants who require immediate therapeutic 18 to regulate motoric agitation and muscle tone
Kumar, N. et al. [12]	“Role of neuroimaging in preterm infants to predict neurological outcomes”	2023	prospective cohort study	India	In total, 56 preterm infants (<34 weeks)	Intracranial complications are significantly associated with lower GA and birth weight; on the contrary, a higher-than- expected incidence of abnormalities (44.4%) was found in infants > 2 kg
McLean, G. et al. [13]	“Evaluation of a Cranial Ultrasound Scoring System for Prediction of Abnormal Early Neurodevelopment in Preterm Infants”	2023	retrospective cohort study	Australia	In total, 242 preterm infants (median GA 26.5 weeks)	A formalized HUS system, including subtle markers (corpus callosum thinning, delayed folding), is more sensitive (57%) for predicting CP than standard “severe abnormality” reporting (27%); late screening (6 weeks postnatal or TEA) is essential to detect WM injury and brain atrophy that are not visible on initial early-life scans
Toma, A.I. et al. [14]	“Correlations between Head Ultrasounds Performed at Term-Equivalent Age in Premature Neonates and General Movements Neurologic Examination Patterns”	2023	prospective cohort study	Romania	In total, 44 preterm neonates (mean GA, 33.59 weeks (+2.43 weeks))	Atypical GM patterns, such as “Poor Repertoire” and “Cramped-Synchronized”, are strongly associated with TEA-HUS markers of white matter volume loss and dysmaturation (reduced basal ganglia width and immature gyration); these structural findings together may be associated with long-term motor deficits
Chevallier, M. et al. [15]	“Decision-making for extremely preterm infants with severe hemorrhages on head ultrasound: Science, values, and communication skills”	2023	literature review	Canada	extremely preterm infants	Bilateral involvement (laterality) and post-hemorrhagic ventricular dilatation are far more reliable predictors of CP than the specific anatomical location of the hemorrhage; the need for surgical shunting significantly worsens the motor prognosis, whereas traditional grading systems have limited utility compared to assessing the total extent of the injury
Mayrink, M.L.D.S. et al. [16]	“The trajectory of head circumference and neurodevelopment in very preterm newborns during the first two years of life: a cohort study”	2024	prospective cohort study	Brazil	In total, 95 newborns (<32 weeks or 1500 g)	HC is a practical, high-value clinical marker for neurodevelopment; higher HC growth at 5 months corrected age correlates positively with cognitive, motor, and language at 18 months; the window between discharge and 1 month corrected age is a critical period for catch-up growth and prediction of functional status
Toma, A.I. et al. [17]	“Cerebral Ultrasound at Term-Equivalent Age: Correlations with Neuro-Motor Outcomes at 12–24 Months Corrected Age”	2024	prospective cohort study	Romania	In total, 34 premature infants (30–34 weeks GA), followed to 24 months	Abnormal motor acquisitions at 24 months correlate with structural markers at TEA, specifically increased ventricular midbody size, decreased basal ganglia width, decreased cortical depth, and immature gyration; the association is strong enough to support the potential development of a simplified clinical scoring system for motor deficit prediction via HUS
Necula, A.I. et al. [18]	“Neurological Outcomes in Late Preterm Infants: An Updated Review of Recent Research and Clinical Insights”	2025	literature review	Romania	late preterm infants	Structural alterations in the posterior region of the corpus callosum are linked to motor integration deficits and cognitive delays that persist into school age

## Data Availability

No new data were created or analyzed in this study. Data sharing is not applicable to this article.

## Abbreviations

The following abbreviations are used in this manuscript:

ADHD	Attention Deficit–Hyperactivity Disorder
NICU	Neonatal Intensive Care Unit
VLBW	Very Low Birth Weight
HUS	Head Ultrasound
IVH	Intraventricular Hemorrhage
MRI	Magnetic Resonance Imaging
PVL	Periventricular Leukomalacia
TEA	Term-Equivalent Age
AI	Artificial Intelligence
CP	Cerebral Palsy
GMs	General Movements
HC	Head Circumference
WM	White Matter

## References

[1] Fletcher J.M., Landry S.H., Bohan T.P., Davidson K.C., Brookshire B.L., Lachar D., Francis D.J. (1997). Effects of intraventricular hemorrhage and hydrocephalus on the long-term neurobehavioral development of preterm very-low-birthweight infants. Dev. Med. Child Neurol..

[2] Davies M.W., Swaminathan M., Chuang S.L., Betheras F.R. (2000). Reference ranges for the linear dimensions of the intracranial ventricles in preterm neonates. Arch. Dis. Child. Fetal Neonatal Ed..

[3] Leijser L.M., Srinivasan L., Rutherford M.A., Counsell S.J., Allsop J.M., Cowan F.M. (2007). Structural linear measurements in the newborn brain: Accuracy of cranial ultrasound compared to MRI. Pediatr. Radiol..

[4] Inder T.E., Wells S.J., Mogridge N.B., Spencer C., Volpe J.J. (2003). Defining the nature of the cerebral abnormalities in the premature infant: A qualitative magnetic resonance imaging study. J. Pediatr..

[5] Mutlu A., Livanelioğlu A., Korkmaz A. (2010). Assessment of “general movements” in high-risk infants by Prechtl analysis during early intervention period in the first year of life. Turk. J. Pediatr..

[6] Toma A.I. (2023). Paediatric neurology: Standardization of neonatal assessment in Romania. Enfance.

[7] Kwong A.K., Eeles A.L., Spittle A.J. (2021). Early neurodevelopmental screening: Parent perspectives from the neonatal intensive care unit. Acta Paediatr..

[8] Guillot M., Sebastianski M., Lemyre B. (2021). Comparative performance of head ultrasound and MRI in detecting preterm brain injury and predicting outcomes: A systematic review. Acta Paediatr..

[9] Inder T.E., de Vries L.S., Ferriero D.M., Grant P.E., Ment L.R., Miller S.P., Volpe J.J. (2021). Neuroimaging of the preterm brain: Review and recommendations. J. Pediatr..

[10] Zhang X.H., Chen W.J., Gao X.R., Li Y., Cao J., Qiu S.J. (2021). Predicting the developmental outcomes of very premature infants via ultrasound classification: A CONSORT-clinical study. Medicine.

[11] Helderman J., O’Shea T.M., Dansereau L., Check J., Hofheimer J.A., Smith L.M., McGowan E., Neal C.R., Carter B.S., Pastyrnak S.L. (2022). Association of abnormal findings on neonatal cranial ultrasound with neurobehavior at neonatal intensive care unit discharge in infants born before 30 weeks’ gestation. JAMA Netw. Open.

[12] Kumar N., Kumar D., Priyadarshi M., Kumar A., Bharti A.K., Nath K.S., Ahmad G.S. (2023). Role of neuroimaging in preterm infants to predict neurological outcomes. Int. J. Acad. Med. Pharm..

[13] McLean G., Razak A., Ditchfield M., Lombardo P., Malhotra A. (2023). Evaluation of a Cranial Ultrasound Scoring System for Prediction of Abnormal Early Neurodevelopment in Preterm Infants. J. Paediatr. Child Health.

[14] Toma A.I., Dima V., Alexe A., Rusu L., Nemeș A.F., Gonț B.F., Arghirescu A., Necula A., Fieraru A., Stoiciu R. (2023). Correlations between head ultrasounds performed at term-equivalent age in premature neonates and general movements neurologic examination patterns. Life.

[15] Chevallier M., Barrington K.J., Church P.T., Luu T.M., Janvier A. (2023). Decision-making for extremely preterm infants with severe hemorrhages on head ultrasound: Science, values, and communication skills. Semin. Fetal Neonatal Med..

[16] Mayrink M.L.D.S., Villela L.D., Méio M.D.B.B., Soares F.V.M., Abranches A.D.D., Nehab S.R.G., Reis A.B.R., Barros L.B.d.P., de Rodrigues M.C.C., Junior S.-C.G. (2024). The trajectory of head circumference and neurodevelopment in very preterm newborns during the first two years of life: A cohort study. J. Pediatr. (Rio J.).

[17] Toma A.I., Dima V., Rusu L., Nemeș A.F., Gonț B.F., Arghirescu A., Necula A., Fieraru A., Stoiciu R., Andrășoaie L. (2024). Cerebral Ultrasound at Term-Equivalent Age: Correlations with Neuro-Motor Outcomes at 12–24 Months Corrected Age. Children.

[18] Necula A.I., Stoiciu R., Radulescu Botica R., Durdu C.E., Bohiltea R. (2025). Neurological outcomes in late preterm infants: An updated review of recent research and clinical insights. Diagnostics.

[19] Lubián-Gutiérrez M., Benavente-Fernández I., Marín-Almagro Y., Jiménez-Luque N., Zuazo-Ojeda A., Sánchez-Sandoval Y., Lubián-López S.P. (2024). Corpus callosum long-term biometry in very preterm children related to cognitive and motor outcomes. Pediatr. Res..

[20] Maitre N.L., Marshall D.D., Price W.A., Slaughter J.C., O’Shea T.M., Maxfield C., Goldstein R.F. (2009). Neurodevelopmental outcome of infants with unilateral or bilateral periventricular hemorrhagic infarction. Pediatrics.

[21] Hintz S.R., Barnes P.D., Bulas D., Slovis T.L., Finer N.N., Wrage L.A., Das A., Tyson J.E., Stevenson D.K., Carlo W.A. (2015). Neuroimaging and neurodevelopmental outcome in extremely preterm infants. Pediatrics.

[22] Ballabh P., de Vries L.S. (2021). White matter injury in infants with intraventricular haemorrhage: Mechanisms and therapies. Nat. Rev. Neurol..

[23] Inder T.E., Anderson N.J., Spencer C., Wells S., Volpe J.J. (2003). White matter injury in the premature infant: A comparison between serial cranial sonographic and MR findings at term. Am. J. Neuroradiol..

[24] Li H., Liu M., Zhang J., Liu S., Fang Z., Pan M., Xu Y., Ge X. (2024). The effect of preterm birth on thalamic development based on shape and structural covariance analysis. NeuroImage.

[25] Kidokoro H., Neil J.J., Inder T.E. (2013). New MR imaging assessment tool to define brain abnormalities in very preterm infants at term. Am. J. Neuroradiol..

[26] Nongena P., Ederies A., Azzopardi D.V., Edwards A.D. (2010). Confidence in the prediction of neurodevelopmental outcome by cranial ultrasound and MRI in preterm infants. Arch. Dis. Child. Fetal Neonatal Ed..

[27] Hintz S.R., Vohr B.R., Bann C.M., Taylor H.G., Das A., Gustafson K.E., Yolton K., Watson V.E., Lowe J., DeAnda M.E. (2018). Preterm neuroimaging and school-age cognitive outcomes. Pediatrics.

[28] Yum S.K., Im S.A., Seo Y.M., Sung I.K. (2019). Enlarged subarachnoid space on cranial ultrasound in preterm infants: Neurodevelopmental implication. Sci. Rep..

[29] Li Y.T., Chen L.W., Koh C.L., Lin Y.C., Huang C.C. (2025). Functional connectivity as a prognostic biomarker for neurodevelopmental outcomes in preterm infants without severe brain injury. Brain Commun..

[30] Cizmeci M.N., de Vries L.S., Ly L.G., van Haastert I.C., Groenendaal F., Kelly E.N., Govaert P., Leijser L.M. (2020). Periventricular hemorrhagic infarction in very preterm infants: Characteristic sonographic findings and association with neurodevelopmental outcome at age 2 years. J. Pediatr..

[31] De Vries L.S., Benders M.J., Groenendaal F. (2015). Progress in neonatal neurology with a focus on neuroimaging in the preterm infant. Neuropediatrics.

[32] Whyte H.E., Blaser S. (2013). Limitations of routine neuroimaging in predicting outcomes of preterm infants. Neuroradiology.

[33] Skiöld B., Hallberg B., Vollmer B., Ådén U., Blennow M., Horsch S. (2019). A novel scoring system for term-equivalent-age cranial ultrasound in extremely preterm infants. Ultrasound Med. Biol..

[34] Sarkar S., Shankaran S., Laptook A.R., Sood B.G., Do B., Stoll B.J., Das A., Guillet R., Higgins R.D., Barks J. (2015). Screening cranial imaging at multiple time points improves cystic periventricular leukomalacia detection. Am. J. Perinatol..

[35] Ibrahim J., Mir I., Chalak L. (2018). Brain imaging in preterm infants <32 weeks gestation: A clinical review and algorithm for the use of cranial ultrasound and qualitative brain MRI. Pediatr. Res..

[36] Hintz S.R., O’Shea M. (2008). Neuroimaging and neurodevelopmental outcomes in preterm infants. Semin. Perinatol..

[37] Legge N., Lutz T., Wocadlo C., Rieger I. (2022). Long term neurodevelopmental outcome of preterm infants with periventricular-intraventricular hemorrhage. J. Paediatr. Child Health.

[38] Dorner R.A., Burton V.J., Allen M.C., Robinson S., Soares B.P. (2018). Preterm neuroimaging and neurodevelopmental outcome: A focus on intraventricular hemorrhage, post-hemorrhagic hydrocephalus, and associated brain injury. J. Perinatol..

[39] Muehlbacher T., Dudink J., Steggerda S.J. (2025). Cerebellar Development and the Burden of Prematurity. Cerebellum.

[40] Drayne J.P., Mella A.E., McLean M.M., Ufkes S., Chau V., Guo T., Synnes A., Miller S.P., Grunau R.E., Weber A.M. (2024). Long-range temporal correlation development in resting-state fMRI signal in preterm infants: Scanned shortly after birth and at term-equivalent age. PLoS Complex Syst..

[41] Guha A., Hunter S.K., Legget K.T., McHugo M., Tregellas J.R. (2026). Greater Increase in Hippocampal Activity During the Early Postnatal Period After Preterm Birth Is Associated with Better Cognitive and Motor Outcomes at 18 Months. Dev. Neurobiol..

[42] Gande N., Bloching M., Hochmayr C., Staudt A., Peglow U.P., Kiechl-Kohlendorfer U., Griesmaier E. (2026). Developmental Trajectories of Very Preterm Infants and Implications for Routine Neurodevelopmental Follow-Up. Acta Paediatr..

[43] Serrano-Gómez M.E., Massó-Ortigosa N., Castellanos-Garrido A.L., Acuña De La Rosa E., García-Barriga V.M., López-Dóriga A., Carballo-Santiesteban G., Guerra-Balic M. (2025). General movements in infants with neurological risk: Associations with motor development and referral patterns for brain magnetic resonance imaging. Children.

[44] Doyle L.W., Spittle A., Anderson P.J., Cheong J.L.Y. (2021). School-aged neurodevelopmental outcomes for children born extremely preterm. Arch. Dis. Child..

[45] Hintz S.R., Newman J.E., Vohr B.R. (2016). Changing definitions of long-term follow-up: Should “long term” be even longer?. Semin. Perinatol..

[46] Duan Y., Sun F.Q., Li Y.Q., Que S.S., Yang S.Y., Xu W.J., Yu W.-H., Chen J.-H., Lu Y.-J., Li X. (2015). Prognosis of psychomotor and mental development in premature infants by early cranial ultrasound. Ital. J. Pediatr..

[47] Hagmann C.F., Robertson N.J., Acolet D., Nyombi N., Ondo S., Nakakeeto M., Cowan F.M. (2011). Cerebral measurements made using cranial ultrasound in term Ugandan newborns. Early Hum. Dev..

[48] Ortega-Leon A., Urda D., Turias I.J., Lubián-López S.P., Benavente-Fernández I. (2025). Machine learning techniques for predicting neurodevelopmental impairments in premature infants: A systematic review. Front. Artif. Intell..

[49] Travis K.E., Lazarus M.F., Scala M., Marchman V.A., Bruckert L., Velasco Poblaciones R., Arnam J.S., Ben-Shachar M., Yeom K.W., Marchuk J.C. (2025). Skin-to-skin holding in relation to white matter microstructure in infants born preterm. Neurology.

[50] Tudor S., Bhatia R., Liem M., Wani T.A., Boyd J., Raza Khan U. (2025). Opportunities and challenges of using artificial intelligence in predicting clinical outcomes and length of stay in neonatal intensive care units: Systematic review. J. Med. Internet Res..

